# Protein-L-Isoaspartyl Methyltransferase (PIMT) Is Required for Survival of *Salmonella* Typhimurium at 42°C and Contributes to the Virulence in Poultry

**DOI:** 10.3389/fmicb.2017.00361

**Published:** 2017-03-07

**Authors:** Pavan K. Pesingi, Manoj Kumawat, Pranatee Behera, Sunil K. Dixit, Rajesh K. Agarwal, Tapas K. Goswami, Manish Mahawar

**Affiliations:** ^1^Division of Veterinary Public Health, Indian Veterinary Research InstituteIzatnagar, India; ^2^Division of Biochemistry, Indian Veterinary Research InstituteIzatnagar, India; ^3^Division of Immunology, Indian Veterinary Research InstituteIzatnagar, India; ^4^Division of Bacteriology and Mycology, Indian Veterinary Research InstituteIzatnagar, India

**Keywords:** isoaspartate, PIMT, *Salmonella*, 42°C, poultry

## Abstract

Poultry birds are asymptomatic reservoir of *Salmonella* Typhimurium (*S*. Typhimurium) but act as source of human infection for this bacterium. Inside the poultry, *S*. Typhimurium experiences several stresses, 42°C body temperature of birds is one of them. Proteins are highly susceptible to temperature mediated damage. Conversion of protein bound aspartate (Asp) residues to iso-aspartate (iso-Asp) is one of such modifications that occur at elevated temperature. Iso-Asp formation has been linked to protein inactivation and compromised cellular survival. Protein-L-isoaspartyl methyltransferase (PIMT) can repair iso-Asp back to Asp, thus enhances the cellular survival at elevated temperature. Here, we show that the *pimt* gene deletion strain of *S*. Typhimurium (Δ*pimt* mutant strain) is hypersensitive to 42°C *in vitro*. The hypersusceptibility of Δ*pimt* strain is partially reversed by plasmid based complementation (*trans*-complementation) of Δ*pimt* strain. Following oral inoculation, Δ*pimt* strain showed defective colonization in poultry caecum, and compromised dissemination to spleen and liver. Interestingly, we have observed three and half folds induction of the PIMT protein following exposure of *S*. Typhimurium to 42°C. Our data suggest a novel role of *pimt* gene in the survival of *S*. Typhimurium at elevated temperature and virulence.

## Introduction

The enteric human pathogen *Salmonella* Typhimurium (*S*. Typhimurium) has worldwide prevalence and is a leading cause of food borne gastroenteritis (Yeung et al., 2014; Khoo et al., 2015). There are two types of *Salmonella* infections, including typhoidal and non-typhoidal (Darwin and Miller, 1999; Gal-Mor et al., 2014). Although typhoid fever caused by *S.* Typhi is more fatal, non-typhoidal *Salmonella* organisms are most common foodborne pathogens. The manifestations of non-typhoidal salmonellosis include mild to moderate gastroenteritis consisting of diarrhea, abdominal cramps, vomiting, and fever (Griffin and McSorley, 2011). The invasive infections can lead to septicaemia (Cohen et al., 1987). Non-typhoidal salmonellosis accounts for about 93.8 million cases with 155,000 deaths annually around the globe (Majowicz et al., 2010).

Among non-typhoidal *Salmonella, Salmonella* Enteritidis (*S.* Enteritidis) and *S*. Typhimurium are most frequently associated serovars with food poisoning in human (Oliveira et al., 2002). While in Europe, *S.* Enteritidis is more prevalent, in USA and India *S*. Typhimurium predominates (Besser et al., 2000; Rahman, 2002; Zhang et al., 2003). *S*. Typhimurium is major invasive non-typhoidal *Salmonella* (iNTS) in countries of Sub-Saharan Africa (Gal-Mor et al., 2014; MacLennan et al., 2014). Human acquire *Salmonella* infection mostly from the contaminated food, such as pork, beef, milk, milk products, poultry meat, eggs, and contaminated water. Poultry meat and eggs are the most common sources of *Salmonella* infections in human (Linam and Gerber, 2007). Poultry birds harbor *S.* Enteritidis and *S*. Typhimurium in their caecum without manifesting any symptoms to very mild enteritis.

To colonize and survive inside the poultry, *Salmonella* must need to defend against various stresses that it encounters inside the body. Along with other stresses (like oxidants, limited availability of nutrients, etc.), high body temperature of birds exert an additional threat to *Salmonella*. *Salmonella* is a mesophilic bacterium that can survive and replicate at a range of temperatures. This suggests the existence of mechanism(s) that can combat temperature stress encountered by *Salmonella*. By decreasing ratio of unsaturated to saturated fatty acids, *S.* Typhimurium modulates fatty acid composition and fluidity of the membrane, a phenomenon that has been correlated with thermotolerance of this bacterium at 45°C (Casadei et al., 2002; Sampathkumar et al., 2004; Álvarez-Ordóñez et al., 2008). Among macromolecules, proteins are the primary targets of temperature mediated inactivation. Temperature mediated modifications include unfolding, aggregation and covalent modifications in the proteins. Chaperones can refold unfolded proteins. Temperature induced expression of chaperones have been reported, suggesting their crucial roles in cellular survival under heat stress (Foster and Spector, 1995; Grimshaw et al., 2003; Waldminghaus et al., 2007). Heat shock protein htrA is shown to be helpful in survival of *S.* Enteritidis in egg white at the body temperature of the poultry (42°C) (Raspoet et al., 2014).

Aspartate (Asp) residues in the proteins have been shown to be prone to stress conditions. Under stress Asp converts into *iso-aspartate* (iso-Asp) that can introduce kink in to the polypeptide and subsequently leads in to unfolding of the proteins. Unfolded proteins are prone to make aggregates which have compromised function(s) and can affect cellular survival. For proper refolding of unfolded proteins, repair of covalently modified amino acid residues, like iso-Asp is required before chaperone function. Protein-iso-aspartyl-methyltransferase (PIMT) can repair iso-Asp back to Asp thus enhances cellular survival under stress conditions. PIMT activity has been found in various bacteria, such as *S.* Typhimurium, *Escherichia coli, Pseudomonas aeruginosa, Klebsiella pneumoniae*, and *Enterobacter aerogenes* (Li and Clarke, 1992). The *pimt* gene knock out strains of *Caenorhabditis elegans* (Khare et al., 2009) and *E. coli* (Hicks et al., 2005) were found to be hypersensitive to various stress conditions such as oxidative stress, methanol and elevated temperature of 43°C.

Earlier, we have shown the importance of *pimt* in the survival of *S*. Typhimurium under oxidative stress and virulence in mice (Kumawat et al., 2016). Here, we hypothesized that PIMT might play an important role in the survival of *S*. Typhimurium under temperature stress and aids the colonization of this bacterium in poultry. First, we deleted *pimt* gene from the poultry isolate of *S*. Typhimurium. Then we evaluated *in vitro* survival of *pimt* gene deletion strain at 42°C and in stationary phase. Subsequently, we assessed the contribution of *pimt* gene in the caecal colonization of *S*. Typhimurium.

## Materials and Methods

### Bacterial Strains and Culture Conditions

Five strains of *S.* Typhimurium, E-2375, E-4231, E-4831, E-5587, and E-5591 were procured from the repository of National *Salmonella* Centre (Veterinary type), Indian Veterinary Research Institute (IVRI), Izatnagar, India. *S*. Typhimurium was cultured in LB broth or on Hektoen enteric (HE) agar plates. The DH5α strain of *E. coli* was grown in LB broth or agar. Antibiotics, kanamycin (50 μg ml^-1^) and ampicillin (100 μg ml^-1^) were included in the medium for selection purposes as and when required. The cultures were grown in a 37°C incubator or in a 37°C shaker incubator at 180 rpm. In few experiments the cultures were exposed to 42°C.

### PCR Confirmation of *typh* and Virulence Associated Genes in E-5591 Strain of *S*. Typhimurium

Presence of the *typh* (*S*. Typhimurium specific) and virulence associated genes in the E-5591 strain of *S*. Typhimurium was confirmed by polymerase chain reaction (PCR). *typh* (Alvarez et al., 2004), *hil*A (Guo et al., 2000), enterotoxin (*stn*) (Makino et al., 1999) and *inv*A (Galan et al., 1992) genes were amplified using PCR.

### Construction of *pimt* Gene Deletion Mutant in E-5591 Strain of *S*. Typhimurium and Its Complementation

The plasmids pKD4, pKD46, pCP20 were a kind gift from Dr. Robert J. Maier, Department of Microbiology, University of Georgia, Athens, GA, USA. Plasmid QE 60 (pQE60) was procured from Qiagen, Hilden, Germany.

The *pimt* gene was deleted as per the protocol described earlier (Kumawat et al., 2016). In brief, kanamycin cassette was amplified from pKD46 using *pimt* deletion primers given in **Table 1** and PCR conditions given in **Table 2**. Then the *pimt* gene was replaced by kanamycin cassette. Following confirmation of deletion, the antibiotic cassette was removed by FLP recombinase. The plasmid based complementation was carried out using pQE60-*pimt* and the protocol described earlier (Kumawat et al., 2016). The mutant and complemented strains were confirmed by PCR (Kumawat et al., 2016) and designated as *Δpimt* and *Δpimt* + pQE60-*pimt* strains, respectively.

**Table 1:** Details of the primers used in current study.

Purpose	Primer name	Sequence (5′–3′)	Size of the product (bp)	Reference
Construction of *pimt* deletion mutant	*pimt* deletion	**F**:GATGTGGTTTCAGACTGGTTAGACAGCGTGGG	1600	Kumawat et al., 2016
		AGTTGGCACGCAGTAGGCTGGAGCTGCTTC
		**R**:CGTTTCGGCTTCATCAGGCGTAAGCGTGGGTG
		TTTGCAGGGCAAACATATGAATATCCTCCTTA
	*pimt* test	**F**:ATGAAGGCTACGTCTCCGTC	872	
		**R**:GTGACGTAAGAACCGTGCAAC

**Table 2:** Polymerase chain reaction (PCR) conditions used for amplification of different genes.

S. No.	Gene/purpose	Initial denaturation (temp/time)	Denaturation (temp/time)	Annealing (temp/time)	Extension (temp/time)	Final extension (temp/time)
1	*pimt* deletion	95°C/5 min	95°C/30 s	42°C/30 s	72°C/1 min	72°C/10 min
2	*pimt* test	95°C/30 s	95°C/30 s	60°C/30 s	72°C/90 s	72°C/10 min

### Survival of *Δpimt* Strain at 42°C

Isolated colonies of wild type, *Δpimt* and *Δpimt* + pQE60*-pimt* strains of *S*. Typhimurium were inoculated and grown in LB broth at 37°C for overnight. The overnight grown cultures were diluted in 250 ml of fresh media at a ratio of 1: 100 (old culture: fresh media) and incubated either at 37°C or at 42°C. Aliquots were collected at different times (0, 6, 12, 24, 48, and 72 h) of incubations and serially diluted in phosphate buffered saline (PBS). The colony forming units (CFUs) were determined by plating the serial dilutions on HE agar plates.

### Effect of 42°C Exposure on Expression of the PIMT Protein in *S*. Typhimurium

Overnight grown cultures of wild type and *Δpimt* strains of *S*. Typhimurium were diluted in fresh medium and exposed to 37 or 42°C for 12 h. Following exposure, the cells were pelleted and suspended in ice cold PBS. The cells were lysed by sonication and unbroken cells were removed by centrifugation at 7500 × *g* for 10 min at 4°C. Total proteins in these samples were estimated by bicinchoninic acid protein assay kit (Thermo Scientific, Rockford, IL, USA) and normalized in all samples. Fifty micrograms of proteins were resolved in a 10% SDS-gel and blotted to polyvinylidene difluoride membrane. Following blocking, the membrane was incubated in rabbit anti-PIMT antiserum. The PIMT- anti-PIMT interaction was determined by incubating the membrane in anti-rabbit IgG conjugated with alkaline phosphatase. The blot was developed in nitroblue tetrazolium and 5-Bromo-4-chloro-3-indolyl phosphate containing buffer. Replica gel loaded with same samples was stained with Coomassie Brilliant Blue (CBB) and served as a loading control.

### Effect of *pimt* Gene Deletion on the Colonization Ability of *S*. Typhimurium in Poultry

All animal experimentations were carried out according to the guidelines of the institutional animal ethical committee (IAEC), IVRI, Izatnagar, India. The protocol was approved by the institutional animal ethical committee (IAEC), IVRI, Izatnagar, India. One day old chicks of the White Leghorn breed were procured from the Central Avian Research Institute (CARI), Izatnagar, India. The birds were maintained in cages as per guidelines of the CARI/IAEC and provided with *ad libitum* feed and water. The birds were screened for the presence of *Salmonella* spp. by examining the cloacal swabs, followed by PCR or serotyping. Briefly, the cloacal swabs were taken in buffered peptone water (BPW) and pre-enriched at 37°C for 6 h on a shaker incubator. The pre-enriched cultures were then diluted in the Rappaport Vassiliadis R 10 (RV-10) enrichment broth at a ratio of 1:100 and incubated at 37°C for 24 h. Following incubation, the cultures were streaked on HE agar plates and incubated at 37°C for overnight. The black centered colonies with greenish margin were selected (at least three colonies from each plate) for urease test. The urease negative colonies were tested by *Salmonella* specific PCR using genus specific *invA* primers (Galan et al., 1992) and/or by serotyping using specific anti-sera (Pavan Kumar et al., 2014). To analyze colonization abilities of different strains, *Salmonella* free chicks (10 per group) were orally infected with various strains of *S.* Typhimurium. Cloacal swabs from these birds were enriched and streaked on HE agar media. The presence of *S.* Typhimurium was confirmed by PCR/serotyping as described above.

*Salmonella* free chicks (18 per group) were orally infected with wild type or Δ*pimt* strain of *S*. Typhimurium (at the dose of 10^9^ CFUs/ bird). Actual CFUs given to the birds were determined by dilution and plating of the inoculum on HE agar plates. The colonies were enumerated following incubation of the plates at 37°C for overnight.

Caecal colonization and bacterial loads in spleen and liver were assessed at weekly intervals for up to 4 weeks. Following dissection of the birds, the caeca were collected and homogenized in 5 ml BPW. The isolation of *Salmonella* from such homogenates was carried out in a similar way as described in above section. The colonies were screened by *S.* Typhimurium specific *typh* primers (Alvarez et al., 2004).

Half of the spleen and 100 mg of liver were aseptically collected in 1 ml sterile PBS, triturated and serially diluted. One hundred microliters of homogenates were spread on HE agar plates and plates were incubated at 37°C for overnight. CFUs were calculated as per spleen or per gram of liver tissue.

## Results

On day five post-inoculation, 60% of the birds infected with E-5591 strain of *S.* Typhimurium were positive for fecal shedding. While in case of E-2375, E-4231, E-4831, E-5587 strain infected birds the shedding was 0, 0, 0, and 40% respectively.

**FIGURE 1:**
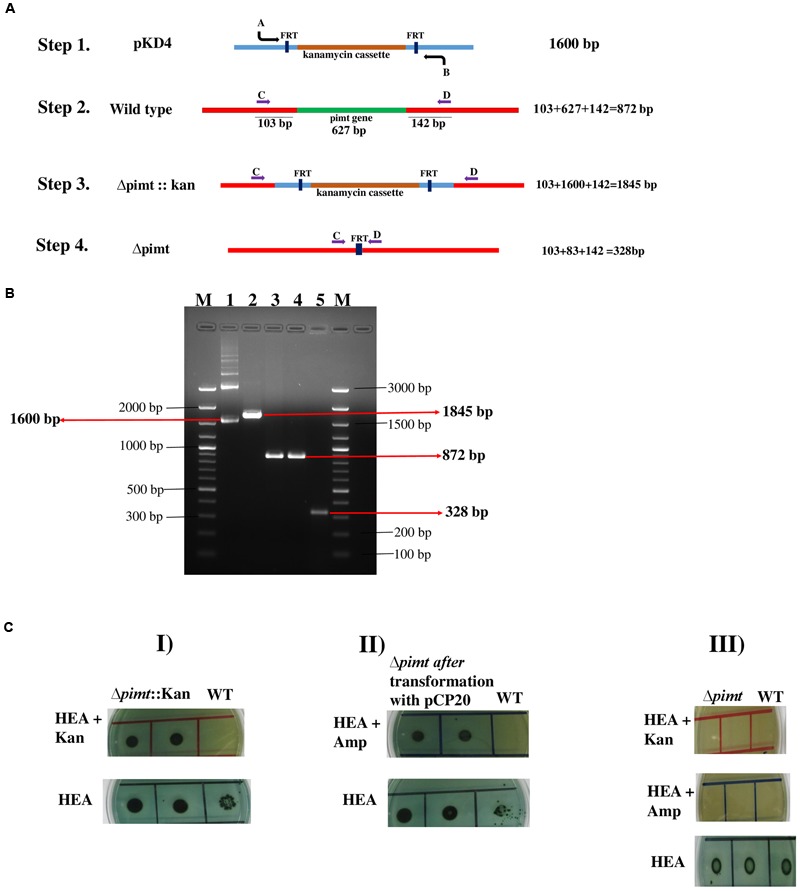
**Construction and confirmation of *pimt* gene deletion strain of *Salmonella* Typhimurium. (A)** Schematics. **(B)** Agarose gel analysis. Kanamycin cassette was amplified and fused to flanking regions of *pimt* gene [step 1 **(A)** and lane 1 **(B)**]. The *pimt* gene was replaced with kanamycin cassette; positive recombinants were selected on kanamycin plate and confirmed by PCR [step 3 **(A)**]. C and D primers amplified bigger and smaller PCR products in mutant and ST colonies respectively (**B**, lanes 2 and 3, respectively). The antibiotic cassette was removed by FLP recombinase; now C and D primers amplified similar sized PCR product in ST colony; while smaller PCR product in case of mutant colony [**(A)** step 4 and **(B)** lanes 4 and 5]. **(C)** Antibiotic resistance patterns observed during course of mutant construction. (I) The *pimt* gene was replaced by kanamycin cassette. Only Δ*pimt*::Kan recombinants were able to grow on kanamycin containing HE agar plates. (II) *Δpimt* mutant after transformation with pCP20. The pCP20 transformed *Δpimt* mutant colonies were able to grow on HE agar-Amp plates. (III) The final *Δpimt* mutant after pCP20 removal. *Δpimt* mutant colonies were unable to grow on antibiotic containing media.

Polymerase chain reaction based analysis confirmed the presence of virulence associated genes (*invA, hilA*, and *stn*) in the E-5591 strain of *S*. Typhimurium (**Supplementary Figure S1**). The schematic used for *pimt* gene deletion has been shown in **Figure 1A**. PCR based analysis confirmed the deletion of *pimt* gene (**Figure 1B**). First recombinants showed kanamycin resistance. After removal of kanamycin cassette the mutant strain failed to grow on antibiotic containing agar plates (**Figure 1C**). Western blot analysis confirmed the presence of PIMT in wild type and complemented strains *S*. Typhimurium, while absence of this protein in Δ*pimt* strain (not shown).

### Effect of 42°C Stress on Survival of Δ*pimt* Strain

**FIGURE 2:**
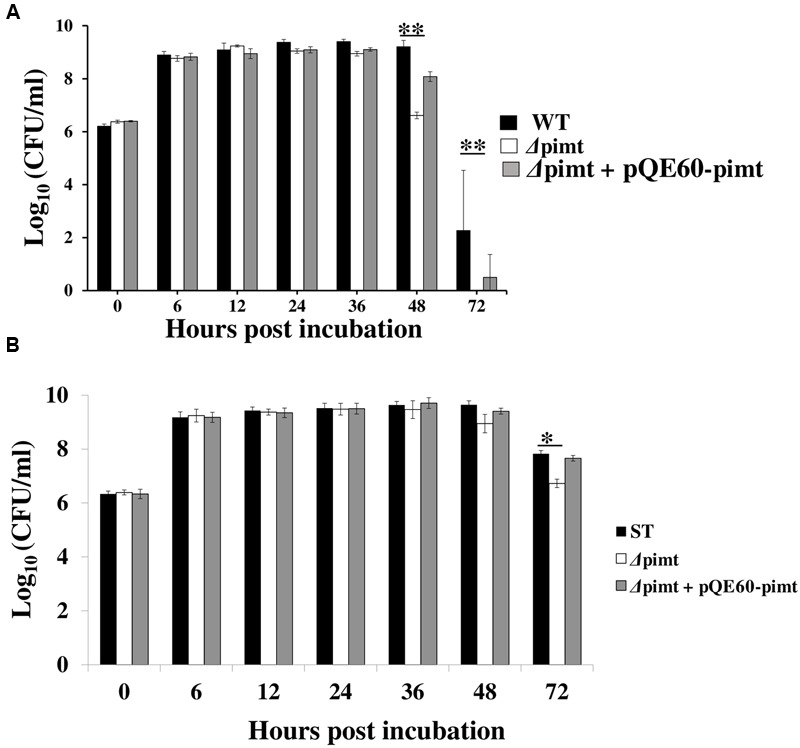
**Effect of heat stress and stationary phase on *in vitro* survival of *Δpimt* strain. (A)**Δ*pimt* strain showed hypersensitivity to 42°C. Overnight grown cultures of WT, *Δpimt* and *Δpimt* + *pQE60-pimt* strains of *S*. Typhimurium were diluted and exposed to 42°C for 72 h in a shaker incubator. The aliquots were drawn at different times post-incubation, serially diluted and plated on HE agar plates. The CFUs/ml were calculated. The data was analyzed by one way ANOVA and presented as mean ± SD (*n* = 4). ^∗∗^*p* < 0.01. **(B)** *Δpimt* strain showed defective survival in stationary phase. Overnight grown cultures of WT and *Δpimt* strains of *S*. Typhimurium were diluted 1: 100 and incubated at 37°C for 72 h. The aliquots were drawn at different times of incubations, serially and plated on HE agar plates. The CFUs/ ml were calculated. The data was analyzed by one way ANOVA and presented as mean ± SD (*n* = 4). ^∗^*p* < 0.05.

We have evaluated the role of *pimt* gene in the survival of *S*. Typhimurium at 42°C *in vitro*. In comparison to wild type, the *Δpimt* strain was highly susceptible (*p* < 0.01, **Figure 2A**) to 42°C exposure for 48 and 72 h. The complemented strain displayed intermediate sensitivity. Following 48 h of incubation, the recoveries were (CFUs/ml as log_10_) 9.20 ± 0.24, 6.61 ± 0.18, and 8.08 ± 0.12 (mean ± SD) for wild type, *Δpimt* and *Δpimt* + pQE60*-pimt* strains of *S*. Typhimurium respectively. Following 72 h of incubation we did not find any viable bacteria in *Δpimt* samples, however, we have recovered significant numbers of live bacteria in wild type and *Δpimt* + pQE60*-pimt* cultures (**Figure 2A**). Interestingly, at 37°C the difference in the viability between wild type and *Δpimt* strains of *S*. Typhimurium was observed at later time point (at 72 h) and was very minor (**Figure 2B**). This indicates that *pimt* is primarily required for survival of *S*. Typhimurium under temperature stress and to a minor extent in late stationary phase.

### Effect of 42°C Exposure on Induction of PIMT Protein

**FIGURE 3:**
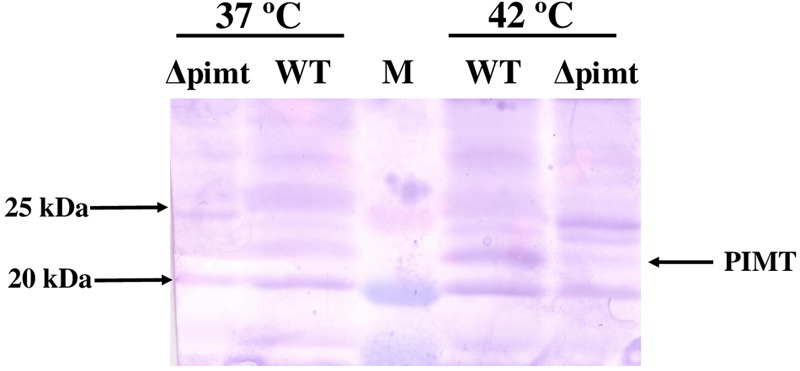
**Effect of heat stress on expression of PIMT protein in *S*. Typhimurium.** Wild type and Δ*pimt* strains of *S*. Typhimurium were grown at 37 or 42°C for 12 h. Cells were then pelleted and induction of PIMT protein was analyzed by SDS-PAGE followed by Western blotting against anti-PIMT antibodies. The incubation temperatures and samples loaded in the lanes are depicted in figure itself. Lane M is protein molecular weight markers. PIMT band is marked by arrow.

After observing the importance of *pimt* gene in the survival of *S*. Typhimurium under temperature stress *in vitro*, we hypothesized that exposure of *S*. Typhimurium to 42°C might induce PIMT protein. Western blot analysis showed a thin band of PIMT in *S*. Typhimurium grown at 37°C, the intensity of which increased about 3.5-fold following exposure of *S*. Typhimurium to 42°C. This band was absent in the Δ*pimt* strain lysate loaded lanes, grown either at 37 or 42°C (**Figure 3**). Replica gel stained with CBB showed similar amounts of protein loading in different lanes (data not shown).

### Role of *pimt* in Caecal Colonization of *S*. Typhimurium

The normal body temperature of poultry is around 42°C (Donkoh, 1989; Cooper and Washburn, 1998) and we have seen hypersusceptibility of Δ*pimt* strain to 42°C *in vitro*. Next we hypothesized that *pimt* might contribute to colonization of *S*. Typhimurium in the poultry. The birds were orally infected with either wild type or Δ*pimt* strain of *S*. Typhimurium and colonization was assessed in the caecum. We recovered bacteria from the 100% caeca of wild type infected birds during entire course (28 days) of experiment. While the caeca of Δ*pimt* strain infected birds showed reduced colonization during first 2 weeks (75 and 60% on days 7 and 14, respectively) and eventually cleared the bacteria on 21 days post-infection (**Table 3**).

**Table 3:** The number of positive/infected birds on different days post-infection.

	*Salmonella* positive birds	
Days (post-infection)	WT inoculated birds	*Δpimt* inoculated birds
7	4/4 (100%)	3/4 (75%)
14	5/5 (100%)	3/5 (60%)
21	4/4 (100%)	0/4 (0%)
28	5/5 (100%)	0/5 (0%)

### Contribution of *pimt* in Dissemination of *S*. Typhimurium to Poultry Spleen and Liver

**FIGURE 4:**
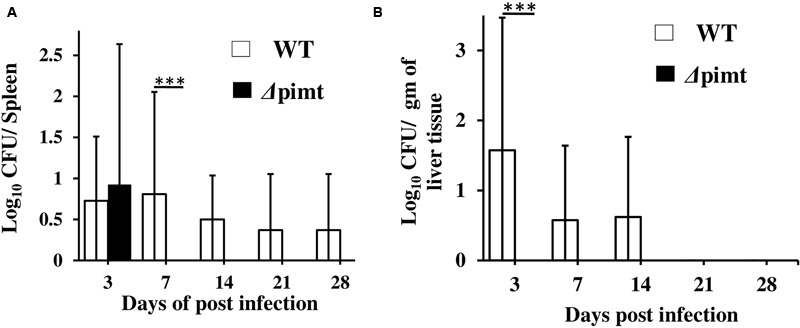
**(A)** Quantification of bacterial burdens in the spleen of the chicks following oral infection with WT or Δ*pimt* strains of *S*. Typhimurium. The spleens were collected, triturated in sterile PBS and 100 μl of such homogenates were plated on HE agar plates. The colonies were counted following incubations of the plates and CFUs/spleen were calculated. The data are presented as mean ± SD, *n* = 5. **(B)** Quantification of bacterial burdens in the liver of the chicks following oral infection with wild type or Δ*pimt* strains of *S*. Typhimurium. The liver tissues were collected, 100 mg of tissue was triturated in sterile PBS and 100 μl of such homogenates were plated on HE agar plates. The colonies were counted following incubations of the plates. CFUs/gram of liver were calculated. The data are presented as mean ± SD, *n* = 5. ^∗∗∗^*p* < 0.001.

The bacterial loads in spleen and liver were determined at different times post-infection. Three days post-infection, we recovered bacteria from the spleens of wild type as well as Δ*pimt* mutant infected birds. However, after 7, 14, and 21 days post-infection, only spleens of wild type infected birds showed bacteria (**Figure 4A**). In liver we observed dissemination of wild type strain of *S.* Typhimurium only. However, we did not recover any bacteria from the liver of Δ*pimt* mutant infected birds at any time post-infection (**Figure 4B**).

## Discussion

Many covalent modifications to amino acid have been described. Conversion of Asp residues to iso-Asp is one of these several covalent modifications that occur in proteins. Iso-Asp formation has been linked with modulation of protein function(s) (David et al., 1999; Lee et al., 2012; Dimitrijevic et al., 2014). PIMT can repair iso-Asp back to normal Asp. Role of *pimt* gene in the survival of various organisms under various stress conditions has been reported (Khare et al., 2009). Recently, we have reported the contribution of *pimt* in the survival of *S*. Typhimurium under oxidative stress and virulence in mice model (Kumawat et al., 2016). In current study, we have evaluated the contribution of *pimt* gene in the survival of *S*. Typhimurium under temperature stress and in stationary phase. Subsequently, we have assessed the contribution of PIMT in the virulence of *S*. Typhimurium in poultry.

First, we procured five strains of *S*. Typhimurium and tested their colonization abilities in the poultry. Based on fecal shedding analysis, E-5591 strain of *S*. Typhimurium showed efficient colonization abilities than other tested strains. After selecting the strain, we constructed *pimt* gene deletion and complemented strains (**Figure 1**).

In comparison to the wild type, the Δ*pimt* strain of *S*. Typhimurium was about 438-fold (*p* < 0.01) more susceptible to 42°C exposure. Complemented strain showed intermediate sensitivity which may be due to expression of PIMT from plasmid pQE-60 in complemented strain (**Figure 2A**). At 37°C the growth of Δ*pimt* strain was similar as of *S*. Typhimurium for up to 36 h. However, the mutant strain (as compared to wild type) showed significant growth difference after 72 h (i.e., in very late stationary phase) but the susceptibility was only 12-fold more than wild type (**Figure 2B**).

Next, we wondered if PIMT protein gets induced following exposure of *S*. Typhimurium to elevated temperature. Interestingly, temperature stress (42°C) has more effect on PIMT induction (**Figure 3**, about 3.5-fold) as compare to oxidative stress as observed in our earlier study (Kumawat et al., 2016, 1.5-fold). Taken together, our current and previous data (Kumawat et al., 2016) suggest that *pimt* is primarily required for *S*. Typhimurium survival against temperature stress and secondarily aids to the survival of this bacterium against oxidants and in stationary phase.

*pimt* gene contributes to the survival of *C. elegans* (Khare et al., 2009), *E. coli* and *Salmonella* Typhimurium (Kumawat et al., 2016) under oxidative stress conditions. Few studies have suggested the importance of *pimt* gene in survival of *E. coli* under heat stress and in stationary phase (Visick et al., 1998). Further, in *E. coli* over expression of PIMT inhibited the aggregation of β**-**galactosidase at 43°C (a protein that aggregates at 43°C), decreases the level of iso-aspartates in this protein, and increases its thermal stability (Kern et al., 2005), suggesting a direct role of PIMT in protection of proteins at elevated temperatures. Interestingly, significantly higher PIMT specific activities were observed following exposure of HeLa cells and *Arabidopsis* at elevated temperatures (Ladino and O’Connor, 1990; Villa et al., 2006).

Oxidative stress and high body temperature (42°C) of the birds are two important stresses that *Salmonella* encounters inside the poultry. We evaluated the colonization abilities of Δ*pimt* strain in poultry. Wild type *S*. Typhimurium colonized in caecum of 100% infected birds. However, Δ*pimt* strain showed some colonization initially but was failed produce a chronic infection (**Table 3**). Similarly, Δ*pimt* showed compromised dissemination to spleen and not at all able to invade liver (**Figure 4**). Taken together, our data suggest that *pimt* contributes to the *S*. Typhimurium virulence in poultry.

Bacteria encode an array of factors which help them to alleviate various environmental and host generated stress that they encounter. A combination of these virulence factors ensures successful bacterial colonization and survival in the host. Protein repair enzymes can serve as one category of such virulence factors. Protein repair enzymes, including PIMT may not act directly as virulence factors. However, by repairing key iso-aspartate residues in proteins (iso-Asp containing proteins), PIMT might be help bacterial survival in the host (heat stress in this case of *S*. Typhimurium). The observed phenotype of Δ*pimt* mutant might be a combination effect of functions of these target genes which required during colonization of *S.* Typhimurium. Therefore, it would be interesting to identify these iso-Asp containing proteins and assess their contribution in the survival of *S.* Typhimurium under heat stress and virulence.

## Author Contributions

PP, TG, RA, and MM designed the experiments. PP, MK, PB, and SD performed all the experiments. PP and MK analyzed the data. PP and MM wrote the paper.

## Conflict of Interest Statement

The authors declare that the research was conducted in the absence of any commercial or financial relationships that could be construed as a potential conflict of interest.

The reviewer LS and handling Editor declared their shared affiliation and the handling Editor states that the process nevertheless met the standards of a fair and objective review.

## Supplementary Material

FIGURE S1 **Polymerase chain reaction confirmation of presence of virulence associated (*stn, hilA*, and *invA*) and Typhimurium specific (*typh*) genes in ST E-5591.** Above mentioned genes were amplified from genomic DNA of *S*. Typhimurium E-5591 and analyzed on 1% agarose gel. PCR products are marked by arrows.
