# Supplementary figures and images for: Protein-L-Isoaspartyl Methyltransferase (PIMT) Is Required for Survival of Salmonella Typhimurium at 42°C and Contributes to the Virulence in Poultry
Source: Front Microbiol. 2017 Mar 7;8:361. doi: 10.3389/fmicb.2017.00361 (PMC5339242; doi:10.3389/fmicb.2017.00361)

## Slide 1
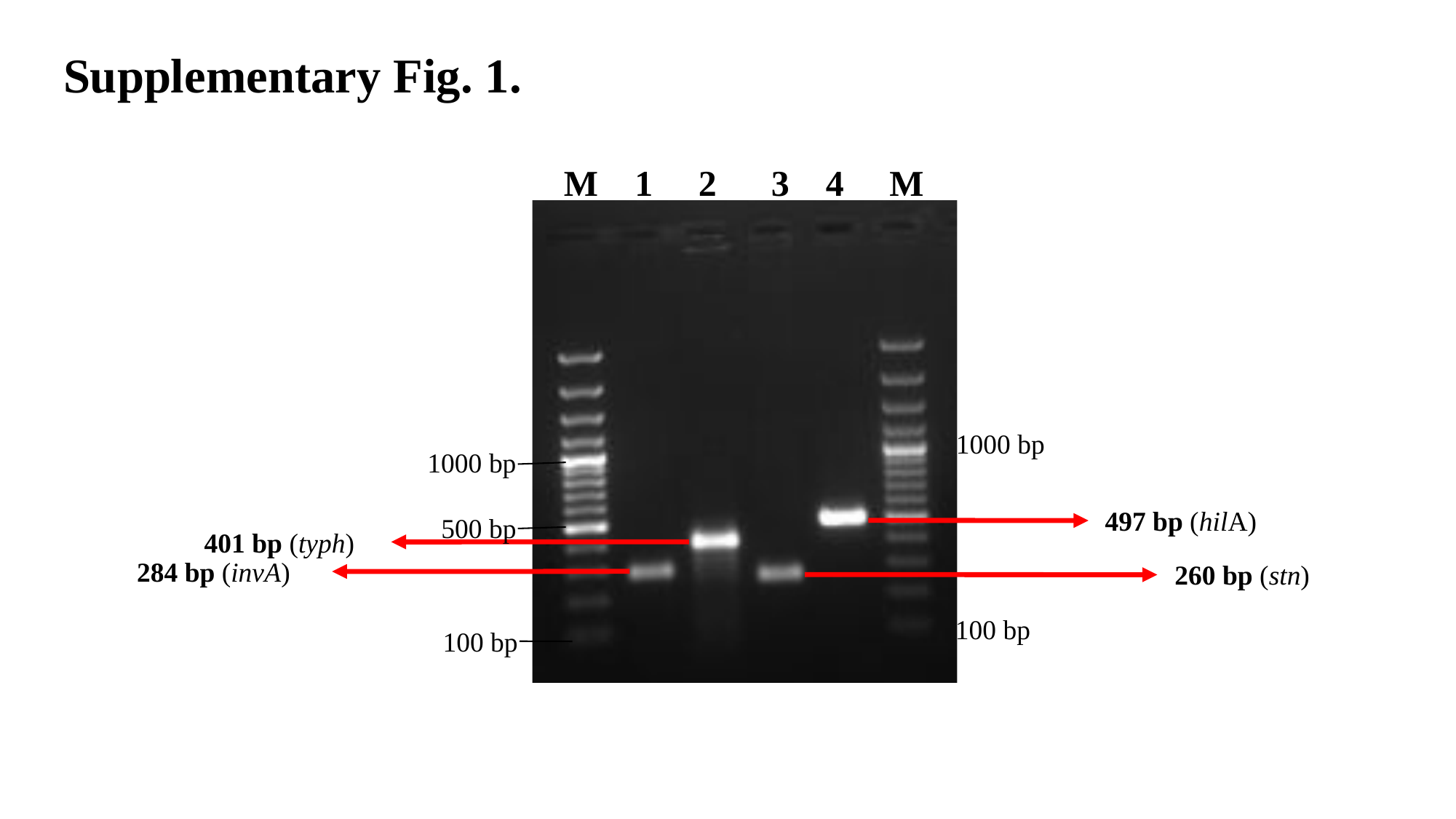

Supplementary Fig. 1.
M 1 2 3 4 M
1000 bp
1000 bp
497 bp (hilA)
500 bp
401 bp (typh)
284 bp (invA)
260 bp (stn)
100 bp
100 bp

Supplement: FIGURE S1 — Polymerase chain reaction confirmation of presence of virulence associated (stn, hilA, and invA) and Typhimurium specific (typh) genes in ST E-5591. Above mentioned genes were amplified from genomic DNA of S. Typhimurium E-5591 and analyzed on 1% agarose gel. PCR products are marked by arrows. [file Presentation_1.ppt]
